# Growth and Bone Mineralization of Very Preterm Infants at Term Corrected Age in Relation to Different Nutritional Intakes in the Early Postnatal Period

**DOI:** 10.3390/nu9121318

**Published:** 2017-12-02

**Authors:** Michelle N. Körnmann, Viola Christmann, Charlotte J. W. Gradussen, Laura Rodwell, Martin Gotthardt, Johannes B. Van Goudoever, Arno F. J. Van Heijst

**Affiliations:** 1 Department of Paediatrics, Subdivision of Neonatology, Radboudumc Amalia Children’s Hospital, Radboud University Medical Center, P.O. Box 9101, Internal Postal Code 804, 6500 HB Nijmegen, The Netherlands; michelle.kornmann@radboudumc.nl (M.N.K.); charlotte.gradussen@radboudumc.nl (C.J.W.G.); arno.vanheijst@radboudumc.nl (A.F.J.V.H.); 2 Department for Health Evidence, Radboud Institute for Health Science, Radboud University Medical Center, 6500 HB Nijmegen, The Netherlands; laura.rodwell@radboudumc.nl; 3 Department of Radiology and Nuclear Medicine, Radboud University Medical Center, 6500 HB Nijmegen, The Netherlands; martin.gotthardt@radboudumc.nl; 4 Department of Paediatrics, VU University Medical Center, 1081 HV Amsterdam, The Netherlands; h.vangoudoever@vumc.nl; 5 Department of Paediatrics, Emma Children’s Hospital—Academic Medical Center (AMC), 1105 AZ Amsterdam, The Netherlands

**Keywords:** calcium, phosphorus, bone mineral density, weight, length, human milk, fortification, preterm formula, dual-X-ray absorptiometry

## Abstract

Preterm infants often have a reduced bone mineral content (BMC) with increased risk of metabolic bone disease. After birth it is difficult to supply calcium (Ca) and phosphorus (P) comparable to the high fetal accretion rate. It is not known whether high supplementation of minerals in the early postnatal period improves growth and bone mineralization. The aim of this study was to evaluate growth and bone mineralization at term corrected age (TCA) in very and extremely preterm infants who received different enteral Ca and P intakes during the first 10 days of life. Infants (*n* = 109) with birth weights below 1500 g were randomly assigned to one of three groups that differed in the nutritional protocols delivered until day 10: Group A, mother’s own milk (MOM) and donor milk (unfortified); Group B, MOM (unfortified) and preterm formula; Group C, MOM (start fortification >50 mL/day) and preterm formula. Due to the earlier commencement of fortification, Group C received higher intakes of calcium and phosphorus and protein (*p* < 0.001) until day 10. At TCA weight, length, BMC and bone mineral density (BMD), measured by dual-X-ray absorptiometry, were not different between the groups. Nutritional intake of P was positively associated with length (β; (95% confidence interval (CI): 0.20 (0.001; 0.393); *p*-value = 0.048), whereas Ca intake was negatively associated with BMC (−1.94 (−2.78; −1.09); *p*-value < 0.001). A small interaction between Ca and P intake was only found for BMD (0.003 (0.00002; 0.00006); *p*-value = 0.036). The volume of human milk per kg provided during the first 10 days was positively associated with BMC (β; (95% CI): 0.013 (0.002; 0.023); *p* < 0.017). Higher intakes of Ca and P during the first 10 days, as provided in this study, did not improve bone mineralization at term corrected age.

## 1. Introduction

Very and extremely preterm infants are known to have a reduced bone mineral content (BMC) with increased risk of development of metabolic bone disease (MBD) [1,2,3,4,5,6]. There are numerous reasons for impaired bone development in preterm infants, but an adequate supply of substrates of calcium (Ca) and phosphorus (P) is a prerequisite for normal bone mineral accretion, whereas vitamin D is essential for the adequate regulation of the mineral homeostasis and bone mineralization [5,7]. Up to 80% of the body Ca of a term infant is accrued during the last trimester of pregnancy [7,8]. Infants born preterm miss this active foetal mineralization in the last trimester, and instead are reliant on supplementation of minerals, provided through parenteral and enteral sources [9,10]. In clinical practice, it is difficult to meet the high foetal needs after preterm birth. Parenteral fluids have a limited solubility for high amounts of Ca and P, whereas human milk has low contents of calcium (Ca) and phosphorus (P) and formula feeding has been shown to have an impaired intestinal absorption of minerals [9,11,12].

Nowadays, it is accepted that early enteral nutrition, and especially human milk (HM), has beneficial health effects. Enrichment of HM with human milk fortifiers (HMF) for preterm infants is the standard of care [13]. However, there is uncertainty with regard to the method of fortification of human milk. The timing and amount of mineral supplementation vary greatly, resulting in varying international practices [14]. Supplementation of Ca and P is often delayed because of fear of nephrocalcinosis, feeding intolerance and necrotizing enterocolitis [15,16]. Early mineral supplementation of human milk at low volumes of enteral intake accelerates the amount of enteral intake, decreases the duration of parenteral nutrition, and may support postnatal growth and bone mineralization in the early postnatal period, whereas delay of fortification may lead to insufficient mineral intake and consecutively impaired bone mineralization. Whether early postnatal high mineral intake will improve bone mineralization has not been evaluated.

The aim of this study was to evaluate bone mineralization and growth at term corrected age (TCA) in very and extreme preterm infants who received either unfortified human milk, preterm formula, or early fortified human milk during the first 10 days of life. We hypothesized that a higher mineral intake would lead to a higher weight and length as well as improved bone mineralization at term corrected age.

## 2. Materials and Methods

### 2.1. Study Design and Randomization

This study (Early Supplementation Study (ESS)) was part of a larger multi-center double-blinded randomized controlled trial: the Early Nutrition Study (ENS) [17]. The ENS evaluated the effects of human milk on postnatal mortality and morbidity, while the ESS evaluated bone mineralization and growth in relation to the timing of mineral supplementation. The studies were approved by the Ethical Committee of the VU University Medical Center, (Amsterdam, The Netherlands) 23 November 2012 (CMO dossier number: NL37296.029.11, Netherlands Trial Registry: NTR 3225). Participants were assigned into one of three groups through two steps of randomization, based on stratification according to birth weight, below or above 1000 g, and appropriate or small for gestational age status. First, infants were randomized into either late mineral and protein supplementation, as part of the ENS (Group A and B), or early supplementation, as part of the ESS (Group C). The second step was only performed if infants were randomized to the late supplementation group. This step randomized infants to either Group A (mother’s own milk (MOM) and/or donor milk) or Group B (MOM and/or preterm formula). Both randomization steps were performed before the first enteral nutrition was administered.

### 2.2. Study Population

Infants were recruited at the level III neonatal intensive care unit of the Radboud University Medical Center (Radboudumc), Nijmegen, Netherlands. Preterm infants, with a birth weight below 1500 g, were eligible for inclusion, if both parents had given written informed consent before the first enteral feeding. Exclusion criteria were congenital malformations, congenital infection proven within 72 h after birth, perinatal asphyxia with a pH <7.0, maternal drugs and/or alcohol use during pregnancy and any intake of cow’s milk based products prior to randomization.

### 2.3. Intervention and Nutritional Protocol

The nutritional protocol and intervention have previously been described [18]. All infants received parenteral nutrition (PN), according to the standard institutional protocol. PN was started directly within the first hour after birth and consisted of standard components with 2.5 mmol/dL calcium gluconate (calcium gluconate 10%; B. Braun, Melsungen, Germany) and 1.6 mmol/dL sodium-glycerophosphate (Glycophos; Fresenius Kabi BV, Zeist, The Netherlands). Table A1 presents the standard protocol for PN. Additional parenteral mineral supplementation with 10% calcium gluconate or sodium-glycerophosphate was administered, according to the discretion of the attending neonatologist, based on blood and urine chemistry.

Enteral feeding, according to group allocation, was started within several hours after birth, with daily increments, while PN was gradually reduced, to maintain daily fluid intake within the protocol range. Where possible, MOM was used for enteral nutrition. If MOM was not available, Group A received donor milk and Group B and C received formula. Preterm formula (Hero Baby Prematuur Start; Hero Kindervoeding, Breda, The Netherlands) contained 2.40 mmol/dL Ca, 1.70 mmol/dL P and 2.6 g/dL proteins. Groups A and B started fortification of human milk or other enteral enrichment only after day 10. For both groups the additional nutritional intake was blinded to all caretakers and parents. Group C received enteral nutrition from day 1 onwards, according to the local protocol. This group received additional enteral supplementation and human milk fortifier (HMF) by the time the enteral intake was 50 mL per day. (Nutrilon Neonatal BMF; Nutriticia, Zoetermeer, The Netherlands) The HMF added 1.65 mmol/dL Ca, 1.22 mmol/dL P and 0.8 g/dL protein. Additional enteral supplementation could comprise of either a supplement of protein (Nutrilon Nenatal protein Fortifier; Nutricia, Zoetermeer, The Netherlands) or a potassium phosphate (KPO_4_) and calcium chloride (CaCl_2_) suspension for enteral supplementation. The decision to start additional enteral supplementation was made by the attending neonatologist and according to the department’s protocol, based on biochemical parameters and postnatal growth.

All infants received vitamin D with parenteral nutrition (80 IE/kg/day) directly after birth. Enteral supplementation was 600 IE (15 micrograms) per day for infants with a weight below 1250 g and 400 IE (10 micrograms) per day for all infants with a weight above 1250 g. Human milk fortifier and preterm formula added 200 IE (5 micrograms)/dL vitamin D; thus, infants received, in total, between 600 and 1000 IE vitamin D per day. According to the local protocol (Group C), vitamin D supplementation by human milk fortification was started. For Groups A and B, enteral vitamin D supplementation was started by day 8 in combination with vitamin K supplementation, according to the national Dutch recommendations.

After 10 days, all infants received nutrition, according to the standard protocol of the Radboudumc, as described above. Around term corrected age (±6 weeks), all surviving participants were invited for an outpatient visit and scheduled for a dual energy X-ray absorptiometry (DXA).

### 2.4. Outcome Measures

All outcome measures were taken up to term corrected age (TCA). Primary outcome measures were bone mineralization and growth. Bone mineralization was measured by dual energy X-ray absorptiometry (DXA), using a whole-body fan beam scanner (Hologic Discovery 85606, software APEX 3.3, Hologic, Vilvoorde, Belgium). Bone mineral content (BMC), bone mineral density (BMD), lean body mass (LBM) and fat mass were determined. Scans showing movement artifacts were classified as unacceptable. Weight and length were determined from the first week onwards, at least weekly, until discharge. Weight was determined using an electronic scale to the nearest 1 g. Crown–heel length was measured to the nearest 5 mm. For participants who had already been transferred, the anthropometric data at TCA were collected from the local hospitals.

### 2.5. Data Registration and Handling

Patient characteristics, clinical course, growth and intake of all nutrients were recorded from the patient records and extracted for this study—daily during the first 14 days and weekly until discharge from the department. After discharge, anthropometric data at TCA were collected from local hospitals. The amounts of enteral, parenteral and additional supplementation (parenteral and enteral) of all nutrients were calculated separately for each patient. For this study, the nutritional intake from the first 10 days was calculated, because this period comprised the intervention period with the maximum difference in nutritional intake. The total intakes were calculated per kg per day for each infant. The intake of nutrients with human milk was calculated using the reference from Gidrewicz et al. [19]. Postnatal growth was evaluated using standard deviation scores (SDS) for weight and length, based on the revised reference chart for preterm infants by Fenton and Kim [20]. Infants with a birth weight below the 10th percentile were classified to be small for gestational age (SGA).

### 2.6. Statistical Analysis

The primary objective of the ESS was to examine whether bone mineralization at TCA differed by type of mineral supplementation at term corrected age (TCA). We performed a power calculation before the enrollment of participants started. We anticipated that a higher intake of minerals would result in a BMC that would, on average, be 5 g higher. Lagemaat et al. found a variability in BMC of 12 g at TCA [21]. Based on two-sided testing with α = 0.05 and β = 0.80, 65 infants per group were required. The statistical analyses were performed using SPSS 22 for Windows (IBM SPSS INC., Chicago, IL, USA). Differences in nutritional characteristics, anthropometric data at TCA and DXA scan measurements were detected using the one-way ANOVA or Kruskal–Wallis test, as appropriate.

The outcomes of interest were two DXA scan measurements, (i.e., BMC and BMD) as well as weight and length. We used Generalized Estimating Equations with an independent correlation structure and robust standard errors to account for the correlation between twins [22]. The stratification factors of birth weight and SGA status as well as gestational age at the time of measurement were included in all analyses. For the primary analysis, separate linear regression models were fitted to examine the association between ESS group and the four outcomes of interest, with Group C being the reference. Continuous predictor variables were centered at their respective means for the analysis. A set of secondary analyses examined the associations between each of the main nutritional variables (i.e., intake of P, Ca, protein per kg per 10 days) and outcomes. An interaction term was included for Ca and P. Finally, the analysis also examined associations between the amount of human milk and the outcomes of interest.

## 3. Results

### 3.1. Patient Characteristics

**Figure 1:**
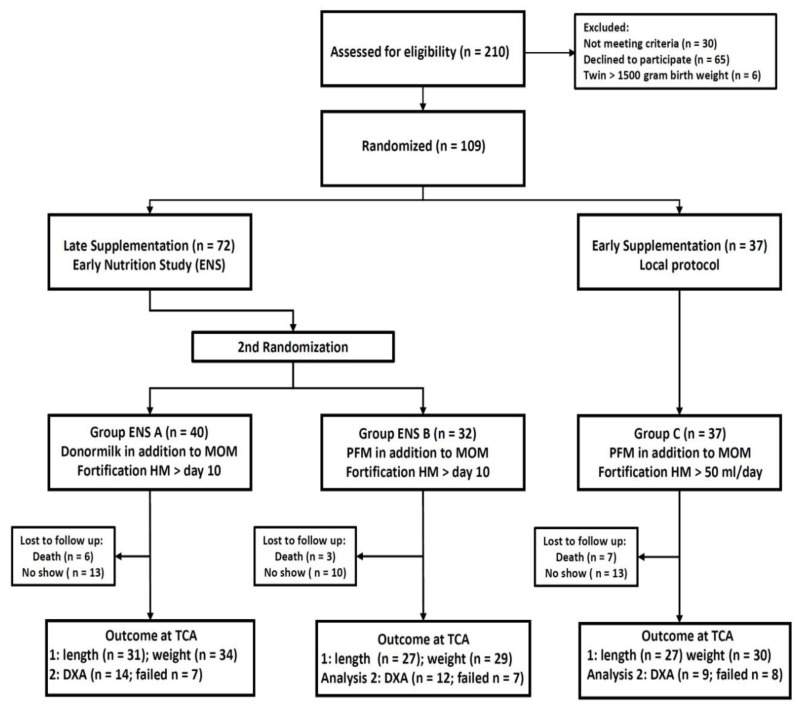
Consort diagram. MOM: mother’s own milk; PFM: preterm formula milk; HM: human milk; TCA: term corrected age; DXA: dual energy X-ray absorptiometry scan.

Patients were enrolled between January 2013 and December 2014. The ENS trial was closed when the required number of infants was included nationwide, forcing us to stop the ESS before the anticipated number of patients was included. The distribution of patients and the exact numbers of measurements are presented in the consort diagram (Figure 1). A total of 109 infants were randomized to either early supplementation (Group C, *n* = 37) or to late supplementation and distributed into Groups A (*n* = 40) and B (*n* = 32). All surviving infants (Group A, *n* = 34, Group B, *n* = 29, Group C, *n* = 30) were included in the growth analyses, but only subsets of those were included in the DXA analyses, either because of parent refusal to attend the follow-up visit, or disapproval of the DXA scan as a result of unacceptable movement artifacts. The baseline characteristics of all patients included and the morbidities and relevant medications of patients who survived to TCA are shown in Table 1. The baseline characteristics, frequencies of morbidities and treatments were comparable between the three groups.

### 3.2. Nutritional Intake

The nutritional characteristics for the first 10 days are presented in Table 2. The number of days with parenteral nutrition and the time to achieve full enteral feeding were not different between the three groups. In agreement with the study protocol, Group C started HMF earlier, at a median (IQR) of day 6.0 (4.0–7.0) compared to Group A (day 11.5 (11.0–12.5)) and Group B (day 12.0 (11.0–13.5)) (*p*-value < 0.001). As a result of the study protocol, Group C received significantly higher mean (SD) intakes of both Ca (Group C: 21.5 (4.8) versus Group A: 15.7 (2.6) and Group B: 17.1 (4.0) mmol/kg per 10 days) and P (Group C: 21.6 (3.7) versus Group A: 16.5 (3.1) and Group B: 16.5 (3.0) mmol/kg per 10 days) during the first 10 days (*p*-value < 0.001). Furthermore, Group C received significantly higher mean (SD) intakes of protein (Group C: 32.1 (5.5) versus Group A: 26.8 (5.0) and Group B: 27.5 (4.3) g/kg per 10 days; *p*-value < 0.001) and carbohydrates (Group C: 114.5 versus Group A: 109.1 (10.7) and Group B: 103.6 (11.5) g/kg per 10 days; *p*-value = 0.02) According to the study protocol, Group A received a significantly higher percentage of human milk during the first 10 days, compared to Group B and Group C (*p*-value < 0.001), although these groups received predominantly human milk as well. The calorie intake differed only slightly between the groups. Table A2 presents the distribution of the route of supplementation of Ca and P. This table demonstrates that differences in mineral intake between the groups were based on enteral intake.

### 3.3. Weight and Length at Term Corrected Age

Table 3 presents the anthropometric data at birth and TCA for all groups, including appropriate (AGA) and small for gestational age (SGA) infants. For both time points, weight and length were similar between the groups. All groups decreased in standard deviation score (SDS) for weight, as well as for length. The decrease in SDS for weight varied between −0.41 and −0.75, and for length between −0.33 and −0.69; for both measurements, there were no significant differences.

### 3.4. DXA-Scan at Term Corrected Age

Table 4 presents the data on body composition, as measured by DXA scan. A total of 35 scans were classified as acceptable. Not all infants were able to visit the outpatient clinic on the scheduled day and for logistic reasons, a number of infants were scanned close before discharge from hospital, therefore the gestational age and weight at the time of the scan varied. Nevertheless, the outcomes were similar across the groups for BMC and BMD with a wide interquartile range.

### 3.5. Determinants of Growth and Bone Mineralization

Table 5 presents the results of the analyses evaluating the effect of being a member of one of the three supplementation groups, and the amount of human milk provided during the first 10 days, on growth and bone mineralization. Groups A and B were analyzed with Group C as the reference. Birth weight, gestational age at measurement and being small for gestational age were significant determinants for all outcomes and thus were used as covariates in all analyses. There was little evidence of an association between the studied groups and the outcomes of weight, length, BMC and BMD. The amount of human milk was associated with a significant effect on BMC, with each mL/kg increase in human milk over 10 days associated with an average increase of 0.013 g (*p*-value = 0.017) in BMC. Length and weight were not associated with intake of human milk.

Table 6 presents the results of the regression analysis for the effects of Ca, P and protein intake per kilogram during the first 10 days on growth, and bone mineralization at term corrected age, of very preterm born infants. Based on the assumption that these three nutrients have a complex interrelationship in the effect on growth and bone mineralization, we analyzed the association with weight, length, BMC and BMD in three models by introducing step-by-step Ca, P and protein, thereby aiming to provide insight into—for example, how the effect of Ca might be influenced by the levels of P. In this analysis, the nutritional intakes of Ca, P and protein for the first 10 days were not associated with weight at term age. Phosphorus intake was positively associated with length, with a significant in increase of 0.19 cm (*p*-value = 0.047) length for each mmol/kg received over the first 10 days. This effect remained after inclusion of protein in the model. Calcium intake was associated with a significant negative effect on BMC. Our analysis showed that each mmol/kg of Ca received over the 10 days was associated with a decrease of 1.21 g of BMC (*p*-value: 0.001). The effect persisted and increased slightly with the introduction of phosphorus and protein in the model. The testing on the interaction of Ca and P over the 10 days intake was negative for all outcomes, except for BMD, which showed a small positive effect (β 0.0003 ; 95% CI (0.00002; 0.0006); *p*-value = 0.036).

## 4. Discussion

This randomized cohort study evaluated the effect of different amounts of Ca and P intakes, during the first 10 days of life, on growth and bone mineralization of very preterm infants. The early stopping of patient inclusions led to lower numbers than the originally anticipated 65 infants per group. Thus, the study may be underpowered to answer the research questions. This study found no differences in weight, length, bone mineral content (BMC) and bone mineral density (BMD) between three different intake groups of very and extremely preterm infants at term corrected age. The regression analysis further showed that group assignment was not associated with the studied outcome measures; however, we found significantly positive associations between P intake and length, as well as the amount of human milk intake and BMC. In contrast, Ca intake was associated with a decrease in BMC, which further decreased after the addition of P and protein in the analysis.

Bone mineralization, at term corrected age, in relation to enteral nutrition of preterm infants has been evaluated in only a few studies during the last three decades. Studies often had an observational design and evaluated more stable infants at a higher gestational age than nowadays treated [1,2,4,24,25,26,27,28,29,30,31]. Eleven studies investigated the effect of either human milk, fortified human milk or various compositions of preterm formulas in randomized studies, leading to varying results [32,33,34,35,36,37,38,39,40,41,42]. Only two studies found an increase in BMC, according to gestational age changes, in combination with high amounts of minerals in preterm formula, while others found the highest weight gain and BMC specifically with preterm formula [2,32,34,35,37,42,43]. Since timing, amount of fortification or composition of formulas differed in all studies as well as the method and timing of scanning (single photon absorptiometry versus dual X-ray absorptiometry), it is difficult to compare these results to our findings. The largest double-blinded randomized study, performed by Faerk et al., did not find an effect of human milk fortification or preterm formula on BMC, compared to unfortified human milk [39]. However, infants fed preterm formula had significantly higher weights at TCA and the amount of supplemented phosphorus was significantly associated with weight at TCA. [39] All infants achieved a BMC below that of healthy term born infants [31,39]. This negative result may be explained by a relatively late timing of fortification, at a mean age of 15 days, and a low amount of fortification of human milk, which was below the ESPGHAN (European Society for Peadiatric Gastroenterology Hepatology and Nutrition) recommendation for enteral intakes of Ca and P [44]. In comparison, we could not demonstrate an association between P, Ca and protein intakes and weight in this study; nevertheless, we found that P and the amount of human milk were positively associated with study outcomes. The differences in outcomes could be explained with the fact that our infants received amounts of minerals within the ESPGHAN recommendations.

According to the nutritional protocol of our hospital, the full recommended intake (including parenteral and enteral intakes) was provided, as soon as possible after birth, aiming at a postnatal growth and bone mineralization comparable to development in utero and to limit a postnatal nutritional deficit, as described in several studies [45,46,47]. This included parenteral mineral supplementation directly after birth, early fortification of human milk and additional supplementation of minerals, based on biochemical parameters. Group C, following the institutional protocol, received a significant higher amount of Ca and P during the first 10 days, compared to Groups A and B, who received no enteral fortifications until day 10. The highest weight gain and bone mineralization could be expected in group C. However, the outcomes of group C compared to Groups A and B were not different, and the total group in comparison to the reference population was still growth retarded. Probably, this may be explained by the short intervention period, with only a few days of significantly different enteral intake. Further, this study included relatively more immature and sicker infants, compared to infants in the previously mentioned trials, probably indicating even higher requirements of minerals for very and extremely preterm infants than currently recommended.

The effect of Ca and P intakes on outcomes seemed contradictory. Calcium intake was associated with a significant negative effect on BMC and non-significant negative effects on all other outcomes, while phosphorus intake had a significant positive effect on length, and a non-significant positive effect on BMC. In general, the effect sizes were small, and BMD was the only outcome that indicated a positive interaction between Ca and P. Again, any interpretation should be performed with caution, since the results may be distorted by the small number of patients investigated with DXA scans. However, an explanation for this phenomenon may be that both minerals are closely related in the formation of bone and shortage of one item may influence the effect of the other mineral. Based on our previous study, we have strong indications that the supply of at least phosphorus was insufficient. For the same cohort of infants evaluated in this study, we reported changes in biochemical parameters for calcium and phosphorus homeostasis, in relation to nutritional intake [18]. Despite a high intake of P, serum P concentrations remained low in all three groups. It was demonstrated that serum P concentrations were significantly associated with amino acid intake, indicating that phosphorus was preferably used for cell metabolism instead of bone mineralization. Hypophosphatemia, in relation to high amino acid intake, has previously been reported in preterm infants and currently is recognized as ‘Placental Incompletely Restored Feeding (PI-Refeeding) syndrome’, caused by an imbalanced nutritional intake of amino acids and phosphorus [18,48,49]. Furthermore, we demonstrated that low gestational age was associated with higher renal excretion of phosphorus, irrespective of nutritional intake. Thus, considering the results of both studies, we speculate that, despite a high intake of minerals in Group C, a high cell metabolism and renal phosphorus wasting prevented adequate availability of phosphorus for adequate bone mineralization and consecutively prevented adequate use of calcium for bone mineralization. Again, this indicated that mineral requirements, to achieve bone mineralization equivalent to term born infants, for the most immature infants, may be higher than currently recommended [13,31].

The method of administration of minerals may have affected the outcomes between groups as well as the results of the regression analysis. As demonstrated in Table A2, the groups differed significantly in the amount of enteral supplementation of minerals, but all groups received more than 50% of the total intake as parenteral supplement. Parenteral nutrients are directly available for metabolism, while supply by the enteral route is also determined by the amount of intestinal absorption [10,50]. The parenteral supplement in our study may have compensated for the low enteral intakes in Groups A and B and may have ameliorated differences in outcomes and the analyses regarding the effects of nutritional intake. In comparison, the study of Faerk et al. did not provide any information on parenteral supplements, although the intervention period (start of supplementation) only started at a mean of 15 ± 7 days [39]. Nowadays, the clinical practice for nutritional support of preterm infants is to provide full parenteral nutrition, including mineral supplementation, shortly after birth. Therefore, the provision of parenteral nutrition in the study of Faerk cannot be excluded and one may speculate that the comparatively positive outcomes of infants who received unfortified human milk in this study could partly be explained by parenteral supplementation of nutrients.

Independent of group assignment, the percentage of MOM was very high in this study. In the studies mentioned previously, which found an improvement in bone mineralization, this was overall related to the use of preterm formula with a high amount of minerals. We did not include a group with exclusively preterm formula, because our general practice is to provide preferably the mother’s own milk. On the other hand, a positive effect of human milk on bone development has previously been reported [51,52]. The positive effect of the amount of human milk on BMC in this study supports our assumption that even for very and extreme preterm infants, it should be possible to achieve adequate bone mineralization at term corrected age, in combination with human milk. Furthermore, this study confirmed earlier findings, that early fortification was well-tolerated [53]. However, this study also demonstrated, that current concepts of mineral supplementation and fortification of human milk are insufficient and need to be further evaluated, while recommendations probably need to be adapted.

This study had several limitations. Firstly, this study did not evaluate the maternal vitamin D status, nor were vitamin D concentrations determined in the participating infants. Epidemiological studies have shown a high prevalence of suboptimal vitamin D concentrations in pregnant and lactating women, even in countries where adequate exposure to sunlight and sufficient dietary intakes may be expected [54,55]. The foetus relies on maternal vitamin D stores, and thus postnatal vitamin D insufficiency may have impaired intestinal calcium absorption directly after birth. However, parenteral vitamin D supplementation was started directly after birth in all infants and enteral supplementation within the first week of life, following the recommendations for very low birth weight infants of the ESPGHAN 2010 [13]. Recently, it was demonstrated that doses of vitamin D, as provided in this study, led to sufficient vitamin D concentrations in very low birth weight infants, in the postnatal period, at 4 weeks of age [56]. The small differences in start day of enteral vitamin D supplementation between Groups A/B and C cannot explain the differences found between the groups. Furthermore, all infants received the same amount of parenteral intake, with relatively high amounts of minerals. Based on blood and urine analyses, it was permitted to provide extra parenteral mineral supplementation during the intervention period. The protocol for parenteral nutrition has been the standard of care at our department for many years and we felt it would be unethical to withhold the standard of care to high risk patients. There was room for a more individualized treatment in relation to enteral supplementation. This possibly has ameliorated the differences between the three groups and subsequently decreased the size of any effect of enteral supplementation. On the other hand, the study protocol reflected current generally accepted clinical practice, with a combined parenteral and enteral nutritional intake. We would like to note that none of our patients developed signs of rickets or fractures and that at follow up, around term age, our patients had achieved a median BMC comparable to 38–39 weeks gestation, according to Lapillone et al. [31]. Up to this date, most studies have been limited to the evaluation of enteral intake. We suggest that further improvements in the combined nutritional supply of parenteral and enteral intake in the early postnatal period are possible and that future trials should include both routes of administration, starting directly after birth. A limitation of our study was the fact that we were unable to include the intended number of patients. This may, on the one hand, have led to overestimation of observed effects; on the other hand, there is a chance of having missed certain effects. It should be noted that we only included three nutrients for the regression analysis, while enteral nutrition, especially human milk, is a complex emulsion and the composition and interactions between various factors that may have impacted on the digestion and absorption of nutrients were not included in the analysis and thus may have been missed.

Despite these limitations, we decided to present our data because there are limited studies available investigating bone mineralization in preterm infants at term age, specifically for the group of very and extremely low gestational age. The specific nutritional needs of this high-risk group need further evaluation. Often studies remain of a small size because it is difficult to include sufficient patients within a reasonable timeframe. The strength of this study is the randomized design and that reporting of data occurred accordingly to the recently proposed standardization of nutrition and growth outcomes [57]. This offers the opportunity to combine our data with studies of a comparable setting, which may add to the significance of the overall results.

## 5. Conclusions

On the basis of this study, there is no evidence that early high mineral intake through early fortification of human milk further improves bone mineralization in combination with high parenteral mineral intake. Mineral intake, according to current recommendations for preterm infants, seems to provide an insufficient amount of minerals to achieve bone mineral content, comparable to term born newborns. The positive effect of human milk on bone mineral content needs to be further evaluated.

## Figures and Tables

**Table 1:** Patient characteristics, morbidity, medication.

**Characteristics (All Infants)**	**Group A (*n* = 40)**	**Group B (*n* = 32)**	**Group C (*n* = 37)**
Gestational age, weeks; mean (SD)	28.3 (2.6)	28.4 (2.6)	28.1 (2.4)
Birth weight; grams; mean (SD)	1002 (275)	1012 (219)	1006 (265)
Small for gestational age; *n* (%)	8 (20)	8 (25)	6 (16)
Male; *n* (%)	21 (53)	20 (63)	18 (49)
Singletons; *n* (%)	25 (63)	24 (74)	17 (46)
Antenatal Steroids; *n* (%)	36 (92)	31 (97)	32 (87)
Cesarean section; *n* (%)	19 (48)	20 (63)	25 (68)
Apgar score (5 min); med (IQR)	7.5 (6.5, 9.0)	8.0 (7.0, 9.0)	7.0 (7.0, 8.0)
Apgar score (5 min); <7; *n* (%)	10 (25)	5 (16)	8 (22)
Death before discharge; *n* (%)	6 (15)	3 (9)	7 (19)
**Morbidities (All Infants Discharged)**	**Group A (*n* = 34)**	**Group B (*n* = 29)**	**Group C (*n* = 30)**
Infant respiratory distress syndrome; *n* (%)	19 (56)	17 (59)	17 (57)
Days of mechanical ventilation; med (IQR)	0 (0, 4.0)	1.0 (0.0, 3.0)	0.0 (0.0, 4.0)
Days of Nasal-CPAP; med (IQR)	22.5 (11.0, 40.0)	29.0 (8.0, 41.0)	22.0 (9.0, 42.0)
Chronic lung disease; *n* (%)	12 (35)	13 (45)	8 (27)
Patent ductus arteriosus; *n* (%)	14 (41)	17 (59)	16 (53)
Intra-ventricular hemorrhage grade ≥2; *n* (%)	5 (14.7)	5 (17)	3 (10)
Sepsis; *n* (%);	8 (24)	8 (28)	10 (33)
Necrotizing enterocolitis; Bell stage ≥2; *n* (%)	0	0	1 (3)
Retinopathy of prematurity; *n* (%)	4 (12)	1 (3)	5 (17)
**Medication (All Infants Discharged)**			
Caffeine; *n* (%)	32 (94)	27 (93)	28 (93)
Diuretics; *n* (%)	9 (27)	10 (35)	6 (20)
Corticosteroids; *n* (%)	4 (12)	2 (7)	3 (10)
Sedation; *n* (%)	7 (21)	8 (28)	8 (27)

Group A: donor milk in addition to mother’s own milk (MOM) and no supplements with enteral feeding until day 10; Group B: preterm formula in addition to MOM and no supplements with enteral feeding until day 10; Group C: preterm formula in addition to MOM and fortifier if intake ≥50 mL/day; SD: standard deviation; med: median; IQR: inter quartile range; small for gestational age: below 10th percentile, according to Fenton et al. [20]; Nasal-CPAP: nasal continuous positive airway pressure; chronic lung disease was defined as oxygen dependency at 36 weeks gestational age; patent ductus arteriosus was classified as need for treatment; sepsis was included if present for >72 h postnatally with positive blood culture; necrotizing enterocolitis was determined with staging, according to Bell [23]; diuretics included single doses of furosemide and maintenance diuretics; sedation included morphine and/or midazolam >24 h.

**Table 2:** Nutritional characteristics during the first 10 days of life.

	Group A (*n* = 34)	Group B (*n* = 29)	Group C (*n* = 30)	*p*-Value
**Characteristics**				
Parenteral nutrition, days; med (IQR)	10.5 (8.0, 13.0)	10.0 (9.0, 14.0)	10.0 (9.0, 21.0)	0.71
Enteral (120 mL/kg/day); day; med (IQR)	10.0 (8.0, 13.0)	9.5 (9.0, 12.0)	10.5 (9.0, 16.0)	0.89
Enteral (150 mL/kg/day); day; med (IQR)	12.0 (9.0, 17.0)	11.0 (10.0, 13.0)	12.0 (10.0, 20.0)	0.89
Start day of HMF; med (IQR)	11.5 (11.0, 12.5)	12.0 (11.0, 13.5)	6.0 (4.0, 7.0)	<0.001
Human milk, mL/kg; mean (SD)	552.8 (248.4)	403.1 (294.5)	432.5 (267.9)	0.06
Formula, mL/kg; med (IQR)	-	33.9 (13.9, 162.9)	12.1 (1.1, 48.6)	<0.001
Percentage human milk; med (IQR)	100 (100.0, 100.0)	82.6 (53.2, 97.6)	97.3 (91.8, 99.9)	<0.001
**Intakes**				
Calcium, mmol/kg; mean (SD)	15.7 (2.6)	17.1 (4.0)	21.9 (4.8)	<0.001
Phosphorus, mmol/kg; mean (SD)	16.5 (3.1)	16.5 (3.0)	21.6 (3.7)	<0.001
Protein; grams/kg; mean (SD)	26.8 (5.0)	27.5 (4.3)	32.1 (5.5)	<0.001
Calories; kcal/kg; mean (SD)	836 (108)	838 (86)	832 (168)	0.60
Carbohydrate; grams/kg; mean (SD)	109.1 (10.7)	103.6 (11.5)	114.5 (18.5)	0.02

Enteral: represents the day infants reached an enteral intake of either 120 or 150 mL/kg/day; HMF: human milk fortifier; Ca: calcium; P: phosphorus. All intakes are presented as the sum of the first 10 days; SD: standard deviation; med: median; IQR: inter quartile range. Missing data: Group A (n:2); Group B (n:4); Group C (n:4).

**Table 3:** Outcome of anthropometry at term corrected age.

Growth at TCA	Group A (*n* = 34)	Group B (*n* = 29)	Group C (*n* = 30)	*p*-Value
Gestational age, weeks	40.1 (39.7, 40.8)	40.3 (39.8, 41.1)	40.1 (39.2, 42.6)	0.126
**Weight (grams)**				
Birth	1050 (834, 1263)	1036 (896, 1211)	1024 (800, 1331)	0.998
Term corrected age (TCA)	2980 (2666, 3328)	3070 (2838, 3625)	3115 (2655, 3700)	0.114
**SDS Weight**				
Birth	−0.22 (−1.26, 0.47)	−0.25 (−1.28, 0.50)	−0.15 (−0.93, 0.51)	0.714
Term corrected age (TCA)	−1.15 (−1.93, −0.40)	−1.00 (−1.84, 0.18)	−0.87 (−1.74, −0.06)	0.630
Difference SDS: Birth—TCA	−0.65 (−1.27, −0.34)	−0.41 (−1.24, 0.18)	−0.75 (−1.42, 0.10)	0.707
**Length (cm)**				
Birth	34.5 (32.9, 36.1)	35.0 (32.5, 37.0)	35.0 (32.3, 37.0)	0.704
Term corrected age (TCA)	48.0 (45.0, 49.0)	48.5 (45.0, 50.5)	49.0 (46.0, 51.5)	0.226
**SDS Length**				
Birth	−1.29 (−1.92, −0.24)	−0.62 (−2.34, 0.22)	−0.66 (−1.14, −0.09)	0.190
Term corrected age (TCA)	−1.37 (−2.34, −0.52)	−1.25 (−2.52, −0.26)	−1.48 (−1.99, −0.62)	0.737
Difference SDS: Birth—TCA	−0.66 (−1.64, 0.56)	−0.33 (−1.17, 0.44)	−0.69 (−1.08, −0.27)	0.870

All data are presented as median and inter quartile range (IQR). TCA: term corrected age; GA: gestational age; SDS: standard deviation score.

**Table 4:** Body composition measured by DXA.

DXA Scan	Group A (*n* = 14)	Group B (*n* = 12)	Group C (*n* = 9)	*p*-Value
Gestational age (weeks)	40.5 (36.7, 44.0)	42.1 (37.6, 45.7)	43.7 (36.5, 45.7)	0.569
Weight (g)	3318 (2407, 4290)	3325 (2343, 3856)	3115 (2533, 3920)	0.966
Length (cm)	48.5 (43.8, 52.8)	48.0 (44.0, 52.1)	49.5 (45.0, 53.0)	0.923
Bone area (cm^2^)	314.1 (263.0, 368.3)	326.7 (254.3, 353.5)	286.6 (256.8, 390.6)	0.988
Bone mineral content (gram)	47.6 (42.0, 66.1)	51.1 (35.4, 65.3)	45.4 (35.3, 63.0)	0.967
Bone mineral density (g/cm^2^)	0.164 (0.147, 0.177)	0.157 (0.138, 0.186)	0.157 (0.138, 0.173)	0.819
Lean body mass (gram)	2862 (2064, 3647)	3164 (2289, 3659)	2576 (2254, 3382)	0.665
Fat mass (gram)	568 (314, 888)	473 (397, 918)	641 (231, 914)	0.922
Fat (%)	16.9 (13.5, 20.4)	15.6 (11.0, 21.5)	16.7 (8.7, 23.5)	0.918

All data are presented as median (IQR); GA: Gestational age; DXA: dual energy X-ray absorptiometry.

**Table 5:** Associations between group assignment and human milk and outcomes at term corrected age.

	Weight		Length		BMC		BMD	
**ESS Group**								
	**β (95% CI)**	***p*-Value**	**β (95% CI)**	***p*-Value**	**β (95% CI)**	***p*-Value**	**β (95% CI)**	***p*-Value**
Group A	−93.3 (−260.0; 75.3)	0.280	−0.42 (−1.54; 0.71)	0.464	4.0 (−1.3; 9.3)	0.140	0.008 (−0.005; 0.020)	0.234
Group B	92.5 (−127.7; 312.8)	0.410	0.24 (−1.09; 1.57)	0.724	0.7 (−6.6; 8.0)	0.855	0.004 (−0.011; 0.019)	0.631
Birth weight	0.13 (−0.21; 0.47)	0.453	0.003 (0.001; 0.005)	0.001	−0.003 (−0.02; 0.01)	0.578	−2.43 × 10^−7^ (−0.0003; 0.00003)	0.987
GA at measurement	120.3 (80.3; 160.4)	<0.001	0.78 (0.51; 1.04)	<0.001	2.8 (2.0; 3.5)	<0.001	0.002 (0.001; 0.004)	0.001
Small for gestational age	−842.0 (−1013.5; −670.5)	<0.001	−3.69 (−5.04; −2.33)	<0.001	−13.1 (−21.7; −4.4)	0.003	−0.015 (−0.029; −0.0002	0.046
Constant	3254.5 (3113.5; 3395.6)	<0.001	48.7 (47.9; 49.6)	<0.001	52.1 (481; 56.2)	<0.001	0.158 (0.149; 0.168)	<0.001
**Human Milk**								
	**β (95% CI)**	***p*-Value**	**β (95% CI)**	***p*-Value**	**β (95% CI)**	***p*-Value**	**β (95% CI)**	***p*-Value**
Human milk	0.1 (−3.3; 0.6)	0.635	0.001 (−0.0001; 0.004)	0.199	0.013 (0.002; 0.023)	0.017	0.0000203 (−3.59 × 10^−6^; 0.0000441)	0.096
Birth weight	0.3 (−0.3; 0.8)	0.343	0.003 (−0.0002; 0.006)	0.066	−0.002 (−0.014; 0.011)	0.771	5.59 × 10^−6^ (−0.00002; 0.00003)	0.680
GA at measurement	1.8 (−14.1; 17.8)	0.821	0.05 (−0.029; 0.133)	0.211	2.7 (2.0; 3.4)	<0.001	0.002 (0.001; 0.004)	0.001
Small for gestational age	−930.0 (−1255.8; −604.0)	<0.001	−4.1 (−6.0; −2.3)	<0.001	−10.6 (−19.1; −2.2)	0.013	−0.008 (−0.023; 0.006)	0.275
Constant	3304.1 (3143.2; 3465.1)	<0.001	48.8 (47.9; 49.7)	<0.001	54.0 (51.2; 56.9)	<0.001	0.163 (0.156; 0.169)	<0.001

Group A and B were analyzed with Group C as the reference; The constant term in the models represents the expected value for Group C, for infants born appropriate for GA and who received the mean intake of nutrients. HM: human milk (mL/kg per 10 days); BW: birth weight (gram/kg per 10 days); GA at measurement: gestational age at measurement in weeks; Small for gestational age: below 10th percentile according to Fenton et al. [20]; 95% CI: 95% confidence interval.

**Table 6:** Associations between nutritional intake and outcomes at term corrected age.

	Model 1		Model 2		Model 3	
Weight	β (95% CI)	*p*-Value	β (95% CI)	*p*-Value	β (95% CI)	*p*-Value
Calcium	1.2 (−16.2; 18.7)	0.893	−12.3 (−38.5; 13.8)	0.355	−22.6 (−51.9; 6.8)	0.131
Phosphorus			19.8 (−8.9; 48.2)	0.177	15.8 (−13.2; 44.6)	0.286
Protein					14.2 (−7.8; 36.3)	0.205
Birth weight	0.2 (−0.2; 0.6)	0.320	0.3 (−0.1; 0.6)	0.160	0.3 (−0.1; 0.7)	0.115
GA at measurement	135.4 (91.3; 179.4)	<0.001	137.4 (95.6; 179.2)	<0.001	138.3 (97.9; 178.8)	<0.001
Small for GA	−890.0 (−1112.0; −668.0)	<0.001	−898.4 (−1113.8; −683.1)	<0.001	−849.0 (−1091.7; −606.2)	<0.001
Constant	3240.1 (3142.3; 338.1)	<0.001	3241.6 (3145.5; 3337.6)	<0.001	3233.2 (3134.2; 3332.2)	<0.001
**Length**						
Calcium	−0.02 (−0.13; 0.10)	0.765	−0.15 (−0.33; 0.27)	0.096	−0.12 (−0.32; 0.07)	0.223
Phosphorus			0.19 (0.002; 0.38)	0.047 ^*^	0.20 (0.001; 0.393)	**0.048**
Protein					−0.04 (−0.17; 0.10)	0.606
Birth weight	0.003 (0.001; 0.006)	0.001	0.004 (0.002; 0.006)	<0.001	0.004 (0.002; 0.006)	<0.001
GA at measurement	0.9 (0.7; 1.1)	<0.001	0.93 (0.7; 1.1)	<0.001	0.93 (0.72; 1.14)	<0.001
Small for GA	−4.1 (−5.8; −2.4)	<0.001	−4.2 (−5.7; −2.7)	<0.001	−4.3 (−5.9; −2.7)	<0.001
Constant	48.5 (48.0; 40.0)	<0.001	48.5 (48.0; 49.0)	<0.001	48.5 (48.0; 49.1)	<0.001
**BMC**						
Calcium	−1.21 (−1.92; −0.50)	0.001	−1.56 (−2.42; −0.71)	**<0.001**	−1.94 (−2.78; −1.09)	**<0.001**
Phosphorus			0.65 (−0.31; 1.61)	0.183	0.56 (−0.30; 1.41)	0.202
Protein					0.57 (−0.09; 1.23)	0.092
Birth weight	0.005 (−0.007; 0.01)	0.431	0.006 (−0.007; 0.020)	0.359	0.009 (−0.003; 0.021)	0.158
GA at measurement	2.99 (2.21; 3.76)	<0.001	3.03 (2.31; 3.75)	<0.001	2.98 (2.28; 3.68)	<0.001
SGA	−11.50 (−17.78; −5.23)	<0.001	−12.1 (−18.01; −6.28)	<0.001	−10.50 (−16.71; −4.30)	0.001
Constant	54.2 (51.4; 57.0)	<0.001	54.3 (51.5; 57.0)	<0.001	54.0 (51.1; 56.9)	<0.001
**BMD**							
Calcium	−0.001 (−0.003; 0.00001)	0.052	−0.001 (−0.003; 0.0004)	0.132	−0.002 (−0.0042; 0.0001)	0.061
Phosphorus			0.0001 (−0.002; 0.002)	0.915	−0.0001 (0.002; 0.002)	0.930
Protein					0.001 (−0.001; 0.003)	0.244
Birth weight	0.00001 (−0.00001; 0.00004)	0.337	0.00001 (−0.00001; 0.00004)	0.354	0.00002 (−0.00001; 0.00005)	0.212
GA at measurement	0.003 (0.001; 0.004)	0.001	0.0029 (0.001; 0.005)	0.001	0.003 (0.001; 0.004)	0.001
Small for GA	−0.01 (−0.023; 0.001)	0.084	0.01 (−0.023; 0.001)	0.093	−0.008 (−0.021; 0.006)	0.261
Constant	0.163 (0.157; 0.169)	<0.001	0.163 (0.157; 0.169)	<0.001	0.163 (0.156; 0.169)	<0.001

BMC: bone mineral content (gram); BMD: bone mineral density (gram/cm^2^)—the nutritional variables reflect the intake of the first 10 days; Ca: calcium intake (mmol/kg per 10 days); P: phosphorus intake (mmol/kg per 10 days); protein intake: (gram/kg per 10 days); GA at measurement: gestational age at time of measurement; Small for GA: small for gestational age; all nutritional variables were adjusted for birth weight, GA at measurement and Small for GA. The constant term in the models represents the expected value for Group C, for infants born appropriate for GA, and who received the mean intake of nutrients. Bold *p*-Values reflect a significant association between one of the investigated nutrients and outcome.

## Appendix A

**Table A1:** Standard parenteral nutritional intake.

	Day 1	Day 2	Day 3	Day 4
Fluid mL/kg/day	80	100	125	150
Carbohydrates grams/kg/day	8	9.6	11.7	13.8
Amino acids grams/kg/day	0.75	1.5	2.25	3
Lipids grams/kg/day	1	2	3	3
Energy Quotient Kcal/kg/day	44	62	82	94
Calcium mmol/kg/day	0.75	1.5	2.25	3.00
Phosphorus mmol/kg/day	0.48	0.96	1.44	1.92

Parenteral nutritional intake based on standardized parenteral solutions [35,53]. For infants below 1000 g, amino acids were additionally added, according to current recommendations [12]. Amino acid solution: Primene (Baxter, the Netherlands); lipid emulsion including vitamins: Clinoleic (20%; Baxter, The Netherlands) or SMOFlipid 20% (Fresenius Kabi; The Netherlands).

**Table A2:** Route of supplementation.

Distribution of Mineral Intake	Group A (*n* = 34)	Group B (*n* = 29)	Group C (*n* = 30)	*p*-Value
**Calcium, mmol/kg**				
Total	15.0 (13.8, 17.5)	15.9 (14.1, 19.2)	22.0 (19.3, 25.5)	<0.001
Enteral	3.9 (2.4, 4.8)	6.0 (2.9, 7.5)	10.3 (4.9, 14.7)	<0.001
Enteral supplement	0	0	0	1.000
Parenteral	12.1 (9.4, 15.1)	10.9 (8.2, 12.5)	12.2 (9.4, 15.2)	0.309
Parenteral supplement	0	0	0	1.000
**Phosphorus, mmol/kg**				
Total	16.4 (14.5, 18.5)	16.3 (15.1, 19.1)	21.6 (19.1, 24.1)	<0.001
Enteral	3.0 (1.7, 3.7)	4.1 (2.5, 5.4)	9.4 (3.6, 14.4)	<0.001
Enteral supplement	0.0 (0.0, 0.3)	0	0.0 (0.0, 3.6)	0.063
Parenteral	12.5 (9.8, 16.3)	11.8 (9.2, 14.9)	11.9 (9.0, 16.8)	0.524
Parenteral supplement	3.3 (1.5, 6.1)	3.1 (1.2, 5.1)	2.1 (0.0, 5.6)	0.498

All values are median (IQR). All intake is presented as the sum of the intake during the first 10 days. Missing values: Group A (n:2); Group B (n:4); Group C (n:3).
